# Modelling West Nile virus transmission risk in Europe: effect of temperature and mosquito biotypes on the basic reproduction number

**DOI:** 10.1038/s41598-017-05185-4

**Published:** 2017-07-10

**Authors:** Chantal B. F. Vogels, Nienke Hartemink, Constantianus J. M. Koenraadt

**Affiliations:** 1 Laboratory of Entomology, Wageningen University & Research, P.O. Box 16, 6700 AA Wageningen, The Netherlands; 2 Theoretical Ecology group, Institute for Biodiversity and Ecosystem Dynamics, University of Amsterdam, P.O. Box 94248, 1090 GE Amsterdam, The Netherlands

## Abstract

West Nile virus (WNV) is a mosquito-borne flavivirus which has caused repeated outbreaks in humans in southern and central Europe, but thus far not in northern Europe. The main mosquito vector for WNV, *Culex pipiens*, consists of two behaviourally distinct biotypes, *pipiens* and *molestus*, which can form hybrids. Differences between biotypes, such as vector competence and host preference, could be important in determining the risk of WNV outbreaks. Risks for WNV establishment can be modelled with basic reproduction number (*R*_0_) models. However, existing *R*_0_ models have not differentiated between biotypes. The aim of this study was, therefore, to explore the role of temperature-dependent and biotype-specific effects on the risk of WNV establishment in Europe. We developed an *R*_0_ model with temperature-dependent and biotype-specific parameters, and calculated *R*_0_ values using the next-generation matrix for several scenarios relevant for Europe. In addition, elasticity analysis was done to investigate the contribution of each biotype to *R*_0_. Global warming and increased mosquito-to-host ratios can possibly result in more intense WNV circulation in birds and spill-over to humans in northern Europe. Different contributions of the *Cx. pipiens* biotypes to *R*_0_ shows the importance of including biotype-specific parameters in models for reliable WNV risk assessments.

## Introduction

During the past years, the arthropod-borne West Nile virus (WNV; family: Flaviviridae), caused repeated outbreaks in humans in Europe^1^. WNV outbreaks have thus far been limited to central, southern, and eastern Europe^2–4^, which is in contrast to the much more severe WNV outbreaks that occurred throughout North America^5^. After the initial introduction of WNV in the United States of America in 1999^6^, the virus spread within a few years from the east to the west coast^7^. Such spread has not been observed in Europe.

WNV is maintained in a natural transmission cycle between ornithophilic mosquitoes and birds, whereas mammals, including humans, are usually dead-end hosts^8^. *Culex (Cx.) pipiens* mosquitoes have been identified as one of the most important vectors for WNV, due to their vector competence, feeding preferences, and high abundance during summer^9–11^. The species *Cx. pipiens* consists of two morphologically identical biotypes, *pipiens* and *molestus*, which show different behaviour^12, 13^. Biotype *pipiens* has a preference for birds, and is therefore thought to be an important enzootic vector for WNV, whereas the *molestus* biotype prefers to feed on mammals^14, 15^. Hybrids between both biotypes are considered important bridge vectors that can transmit WNV from birds to humans, due to their more generalist feeding character^9, 10, 14^.

With regard to the situation in northern Europe, it has been shown that European jackdaws and carrion crows, originating from The Netherlands, are susceptible hosts for WNV^16, 17^. Our previous studies showed that northern European *Cx. pipiens* mosquitoes are competent vectors for WNV, and that temperature is an important limiting factor for WNV transmission^18–20^. The presence of susceptible hosts and mosquitoes that under favourable climatic conditions are competent vectors for WNV, suggests that the possibility of WNV transmission in northern Europe cannot be ruled out. Recently, we showed that vector competence of northern European *Cx. pipiens* biotypes and hybrids is differentially affected by temperature^20^. Thus far, differentiation between the *Cx. pipiens* biotypes and hybrids has not been taken into account in WNV risk assessments. However, differences between biotypes in terms of vector competence response to temperature and in terms of feeding preferences are likely to affect their vectorial capacity for WNV. Therefore, this information needs to be taken into account when assessing risks of WNV transmission in northern Europe.

One way to assess the risk of WNV outbreaks is by calculating the basic reproduction number (*R*_0_). The *R*_0_ represents the average number of secondary cases that can arise after introduction of one infectious individual in a susceptible population^21, 22^. If the average number of secondary cases is higher than one (*R*_0_ > 1), there is a risk for disease establishment in a certain area. This risk of disease establishment increases with higher numbers of secondary cases. If the number of secondary cases is lower than one (*R*_0_ < 1), an introduced case may lead to a few new cases, just by chance, but the disease is not expected to establish or cause a large outbreak. Whereas the effect of temperature on the *R*_0_ for WNV has been investigated^23, 24^, no studies differentiated between the behaviourally distinct *Cx. pipiens* biotypes in *R*_0_ models. Differentiation between biotypes is highly important because differences in vector competence and different host feeding behaviour can strongly impact the outcome of *R*_0_ models^14, 20^. Here, we investigated the effects of both temperature and behavioural differences between the *Cx. pipiens* biotypes on *R*_0_, with a focus on the northern European situation. We developed an *R*_0_ model with temperature-dependent and biotype-specific parameters that were mostly determined for European mosquitoes. Based on this model, differences in establishment of WNV transmission cycles across Europe can be explored.

## Methods

### Model description

*R*_0_ is calculated using the next-generation matrix method^21, 25^. We will first derive the next-generation matrix (NGM) and then describe how we can use it to study different scenarios. Each scenario is based on a different combination of parameter values for four factors: temperature, mosquito-to-host ratio, the fraction of birds in the host population, and the biotype composition of the mosquito population.

First, we need to determine the number of types-at-infection. These types-at-infection are the types of individuals that are involved in transmission and that differ from each other from an epidemiological perspective. In this case, there are four types to be distinguished; three mosquito biotypes (biotype *pipiens*, biotype *molestus*, and hybrids) and one (generic) reservoir host type (birds). Non-competent hosts, such as humans and mammals, are not explicitly included in the matrix, as they do not contribute to transmission. However, the fact that non-competent hosts may receive bites by infected mosquitoes and that these bites result in a loss from the perspective of the virus, is taken into account (see section: transmission from bird to mosquito).

The NGM is then a four by four matrix and its elements represent the reproduction numbers for all pairs of types. That is, each element of the matrix, *k*_*ij*_, gives the number of individuals of type *i* that are infected by one newly infected individual of type *j* over the course of its infective lifetime.

We assume that transmission only occurs between birds and mosquitoes, not directly among birds or among mosquitoes. This means that we have to derive three terms for transmission from birds to each of the three biotypes (*k*_pb_, *k*_mb_, *k*_hb_), where subscripts p indicates biotype *pipiens*, m indicates biotype *molestus*, h indicates hybrid, and b indicates bird. Vice versa, we need three terms to describe transmission from each of the biotypes to birds (*k*_bp_, *k*_bm_, *k*_bh_). The NGM can then be specified as follows:

1 $$[\begin{array}{cccc}0 & 0 & 0 & {k}_{{\rm{pb}}}\\ 0 & 0 & 0 & {k}_{{\rm{mb}}}\\ 0 & 0 & 0 & {k}_{{\rm{hb}}}\\ {k}_{{\rm{bp}}} & {k}_{{\rm{bm}}} & {k}_{{\rm{bh}}} & 0\end{array}]$$

Before we derive the terms in the NGM, we here give some notes on the notation. Throughout the manuscript, subscripts are used to indicate temperature- and biotype-specificity of parameters. Subscript x is used to indicate that a parameter value is biotype-specific (but when referring to a specific biotype, we use subscripts p, m, and h, as indicated above). Subscript T indicates that a parameter value is temperature-dependent and the temperature values indicate that it refers to a specific temperature (e.g. EIP_18_ refers to the duration of the extrinsic incubation period at 18 °C).

### Transmission from mosquito to bird

The expected number of birds infected by one newly infected mosquito results from the multiplication of four items:

1. The probability that this newly infected mosquito survives long enough to become infectious, that is, to survive the extrinsic incubation period (EIP). This probability can be estimated in several ways^26, 27^. Here we assume that a vector can only become infectious after completion of the EIP, hence the daily survival probability (*p*) is raised to the power of the duration of the EIP. Both *p* and EIP are temperature-dependent (hence they are indicated by *p*_T_ and EIP_T_).
2. The number of bites per day that this mosquito takes on birds after becoming infectious, which is a multiplication of the temperature-dependent biting rate *a*_T_ (the number of bites per day, or the reciprocal of the number of days between blood meals), and the probability (*ϕ*_x_) that a bite will be on a bird. The modelling of the biotype-specific host preference is described below (section: biotype-specific host preferences *ϕ*_x_).
3. The probability per bite to transmit the virus (*b*).
4. The duration of the infectious period of the mosquito, which is the expected remaining life span of the mosquito (1/-ln(*p*)).

The expected number of birds, infected by one newly infected mosquito is then:

2 $${k}_{{\rm{bx}}}=\frac{{\varphi }_{{\rm{x}}}\ast {a}_{{\rm{T}}}\ast b\ast {p}_{{\rm{T}}}^{{{\rm{EIP}}}_{{\rm{T}}}}}{(-\,\mathrm{ln}({p}_{{\rm{T}}}))}$$

where *a*, EIP and *p* depend on temperature (Table 1).

**Table 1:** Parameter estimates for basic reproduction number (*R*_0_) model.

Parameter	Description	Point Estimates (range)	References
*a*_18_	Daily biting rate at 18 °C	0.14 (0.12–0.16)	^41, 62^
*a*_23_	Daily biting rate at 23 °C	0.17 (0.15–0.19)	^41, 62^
*a*_28_	Daily biting rate at 28 °C	0.20 (0.17–0.23)	^41, 62^
*b*	Transmission probability mosquito to bird	0.80	^24, 63^
*c*_p18_	Transmission probability bird to *pipiens* at 18 °C	0.04 (0.00–0.07)	^20^
*c*_p23_	Transmission probability bird to *pipiens* at 23 °C	0.09 (0.01–0.17)	^20^
*c*_p28_	Transmission probability bird to *pipiens* at 28 °C	0.34 (0.20–0.48)	^20^
*c*_m18_	Transmission probability bird to *molestus* at 18 °C	0.09 (0.01–0.17)	^20^
*c*_m23_	Transmission probability bird to *molestus* at 23 °C	0.13 (0.03–0.22)	^20^
*c*_m28_	Transmission probability bird to *molestus* at 28 °C	0.13 (0.03–0.22)	^20^
*c*_h18_	Transmission probability bird to hybrid at 18 °C	0.05 (0.00–0.11)	^20^
*c*_h23_	Transmission probability bird to hybrid at 23 °C	0.04 (0.00–0.07)	^20^
*c*_h28_	Transmission probability bird to hybrid at 28 °C	0.17 (0.06–0.27)	^20^
1/r_b_	Number of days a bird remains infectious	5.50	^16, 17^
EIP_18_	Extrinsic incubation period at 18 °C (days)	15 (12–18)	^64^
EIP_23_	Extrinsic incubation period at 23 °C (days)	9 (7–11)	^18, 64^
EIP_28_	Extrinsic incubation period at 28 °C (days)	4 (3–5)	^64^
*p*_18_	Daily survival probability at 18 °C	0.97 (0.96–0.98)	^42, 65^
*p*_23_	Daily survival probability at 23 °C	0.96 (0.95–0.97)	^42, 65^
*p*_28_	Daily survival probability at 28 °C	0.95 (0.94–0.96)	^42, 65^
*ϕ*_p_	Host preference *pipiens*	1-(1-FB)^2.75^	^14, 15^
*ϕ*_m_	Host preference *molestus*	FB^2.75^	^14, 15^
*ϕ*_h_	Host preference hybrid	FB	^14, 15^
T	Temperature (°C)	18, 23, or 28	
*C*	Mosquito-to-host ratio	10, 100, or 250	^28^
FB	Fraction of birds in the host population	0.2 or 0.8	
*m*_p_	Fraction *pipiens* in the population	0.10, 0.33, or 0.80	^31, 32^
*m*_m_	Fraction *molestus* in the population	0.10, 0.33, or 0.80	^31, 32^
*m*_h_	Fraction hybrids in the population	0.10 or 0.33	^31, 32^

### Biotype-specific host preferences (*ϕ*_x_)

**Figure 1:**
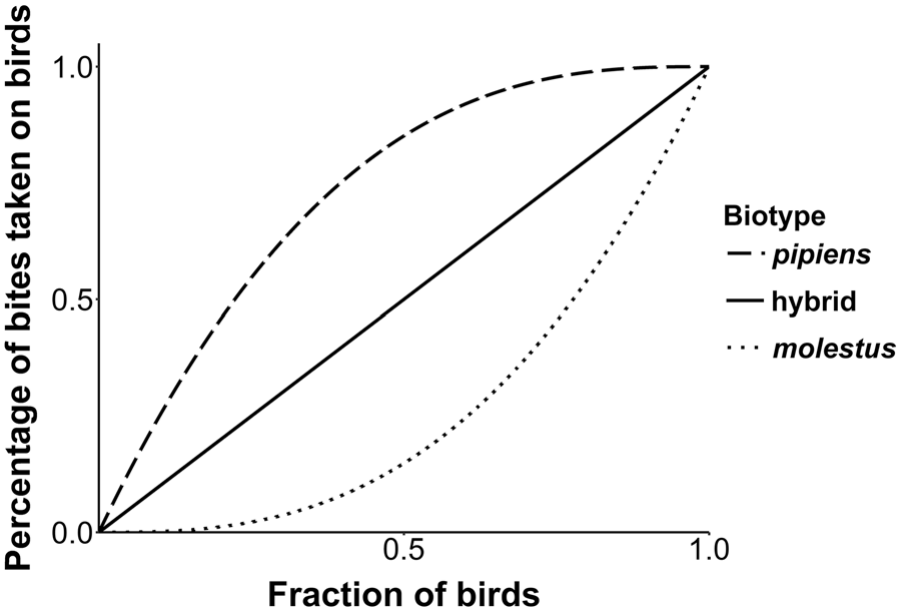
Relation between fraction of birds in the host population and the percentage of bites taken on birds. Percentage of mosquito bites taken on birds by biotype *pipiens*, biotype *molestus*, and hybrids, as a function of the fraction of bird hosts in the total host population.

Each biotype has a different host preference that needs to be taken into account in the model. For each of the biotypes, we derive a simple algorithm that describes how *ϕ*_x_, the probability that a blood meal is taken on a bird, varies with the fraction of birds in the total host population (Table 1 and Fig. 1). We assume that when preferred hosts are not available, mosquitoes will feed on a non-preferred host, rather than not feeding at all. Experimental data indicate that when given a choice between a bird and a mammal, the probability that the mosquito will choose the bird, is approximately 85% for the *pipiens* biotype, 15% for the *molestus* biotype, and 50% for the hybrid^14^. For all biotypes, the probability to feed on a bird when there are no birds should be zero (fraction of birds in the host population (FB) = 0), and the probability is one when there are only birds (FB = 1). For biotype *pipiens*, the function that describes the relation between FB and *ϕ*_x_ should be convex. At equal fractions of birds and other hosts (FB = 0.5), the probability should be approximately 85%. For biotype *molestus*, the function is concave, with a probability to bite a bird of 15% at equal fractions of birds and other hosts (FB = 0.5). For hybrids, the function is a straight line, so that the probability to feed on a bird increases linearly with the fraction of available hosts that is a bird (Fig. 1).

### Transmission from bird to mosquito

The expected number of mosquitoes of a certain biotype infected by one newly infected bird results from the multiplication of:

1. The number of bites that this bird receives from this particular biotype. This number depends on the abundance of the mosquito biotype, its host preference, the number of birds and other hosts, and the temperature-dependent biting rate *a*_T_. Imagine a local mosquito population of *X* individuals. Of these *X* mosquitoes, *a*_T_*X will be searching for a blood meal on a particular day. Multiplying these *a*_T_*X individuals with the fraction of mosquitoes that is of the specific biotype (*m*_p_, *m*_m_ and *m*_h_, for the fractions *pipiens*, *molestus* and hybrid, respectively) and with their respective host preferences *ϕp*, *ϕm*, and *ϕh* gives the number of bites by a certain biotype per bird:
  3 $$({\varphi }_{{\rm{x}}}\ast {a}_{{\rm{T}}}\ast {m}_{{\rm{x}}}\ast X)/{\rm{bird}}$$
  Rather than varying the numbers of mosquitoes, birds and alternative hosts, which would require varying three factors, we prefer to work with two ratios: the mosquito-to-host ratio (*C*), and the fraction of the host population that consists of birds (FB). If the total number of hosts is *Y*, then the number of birds is FB*Y. The mosquito-to-host ratio *C* is equal to *X*/*Y*, so the mosquito-to-bird ratio is *X*/(FB*Y) = *C*/FB. The term for the number of bites of mosquitoes of biotype *x* on birds then becomes:
  4 $${\varphi }_{{\rm{x}}}\ast {a}_{{\rm{T}}}\ast {m}_{{\rm{x}}}\ast C/{\rm{FB}}$$
  For our model, we selected three values for mosquito-to-host ratio based on results of trap catches^28, 29^, which reflect areas with low, intermediate, and high mosquito-to-host ratios (*C* = 10, 100, and 250). In addition, we analysed two values for bird fraction in the host population: low (FB = 0.2) and high bird fraction (FB = 0.8).
2. The biotype- and temperature-specific probability of transmission from bird to mosquito per bite, denoted by *c*_xT_.
3. The expected duration of the infectious period of a bird, which is modelled as the reciprocal of the recovery rate *r*_b_.

The term for the number of mosquitoes of biotype *x* that is infected by a newly infected bird is then as follows:

5 $${k}_{{\rm{xb}}}=\frac{{\varphi }_{{\rm{x}}}\ast {a}_{{\rm{T}}}\ast {c}_{{\rm{xT}}}\ast {m}_{{\rm{x}}}\ast C/{\rm{FB}}}{{r}_{{\rm{b}}}}$$

### Biotype-specific and temperature-dependent transmission efficiency (*c*_xT_)

The probability that a mosquito establishes an infection after feeding on an infected bird generally increases with temperature. However, our earlier work demonstrated that the relationship between temperature and transmission probability is different for each of the three biotypes^20^. We, therefore, used the results of our laboratory experiments on the vector-competence of the three biotypes at 18 °C, 23 °C, and 28 °C^20^. For each biotype, we calculated the confidence interval of the observed values for the vector competence at each temperature (Table 1).

### Scenarios

Based on the terms derived above for the transmission for each of the pairs in the matrix, the NGM now looks as follows:

6 $$[\begin{array}{cccc}0 & 0 & 0 & \frac{{\varphi }_{{\rm{p}}}\ast {a}_{{\rm{T}}}\ast {c}_{{\rm{pT}}}\ast {m}_{{\rm{p}}}\ast C/{\rm{FB}}}{{r}_{{\rm{b}}}}\\ 0 & 0 & 0 & \frac{{\varphi }_{{\rm{m}}}\ast {a}_{{\rm{T}}}\ast {c}_{{\rm{mT}}}\ast {m}_{{\rm{m}}}\ast C/{\rm{FB}}}{{r}_{{\rm{b}}}}\\ 0 & 0 & 0 & \frac{{\varphi }_{{\rm{h}}}\ast {a}_{{\rm{T}}}\ast {c}_{{\rm{hT}}}\ast {m}_{{\rm{h}}}\ast C/{\rm{FB}}}{{r}_{{\rm{b}}}}\\ \frac{{\varphi }_{{\rm{p}}}\ast {a}_{{\rm{T}}}\ast b\ast {p}_{{\rm{T}}}^{{{\rm{EIP}}}_{{\rm{T}}}}}{(-\,\mathrm{ln}({p}_{T}))} & \frac{{\varphi }_{{\rm{m}}}\ast {a}_{{\rm{T}}}\ast b\ast {p}_{{\rm{T}}}^{{{\rm{EIP}}}_{{\rm{T}}}}}{(-\,\mathrm{ln}({p}_{T}))} & \frac{{\varphi }_{{\rm{h}}}\ast {a}_{{\rm{T}}}\ast b\ast {p}_{{\rm{T}}}^{{{\rm{EIP}}}_{{\rm{T}}}}}{(-\,\mathrm{ln}({p}_{{\rm{T}}}))} & 0\end{array}]$$

The value for *R*_0_ can then be calculated for different parameterizations of the NGM. For this purpose, we used the *popbio* package in R to calculate the eigenvalue of the matrix, which represents the *R*_*0*_ estimate^30^. We calculated *R*_0_ based on the parameter ranges given in Table 1, but with different values for:

1. Temperature (T = 18 °C, 23 °C, and 28 °C),
2. Mosquito-to-host ratio (*C* = 10, 100, and 250)^28, 29^,
3. Fraction of hosts that is a bird (FB = 0.2 and 0.8),
4. The biotype composition of the mosquito population, which has been shown to be highly variable across different locations in Europe^31, 32^. To express the variability we selected three representative biotype compositions; either dominated by *pipiens* (*m*_p_ = 0.8, *m*_m_ = 0.1, and *m*_h_ = 0.1), dominated by *molestus* (*m*_p_ = 0.1, *m*_m_ = 0.8, and *m*_h = _0.1) or equal ratios of the three biotypes (*m*_p_ = 0.33, *m*_m_ = 0.33, and *m*_h_ = 0.33).

*R*_0_ values were calculated for each combination of the above-mentioned parameter values, so for a total of (3*3*2*3 = ) 54 different scenarios. For each scenario, we calculated 100,000 *R*_0_ values based on uniform sampling from the parameter value ranges (Table 1). These 100,000 values were then used to calculate the mean *R*_0_ values for each scenario. For each scenario this gives a range of *R*_0_ values rather than a single point estimate, and this range reflects the uncertainty in the parameter estimates.

When interpreting the results, it should be noted that not all scenarios are equally likely to occur, as mosquito biotypes are likely to live in environments where their preferred hosts are found. We hypothesize that dominance of the *pipiens* biotype is most likely to occur in environments where birds are the most abundant hosts (i.e. FB is high) and not very likely in an environment with mostly other hosts (i.e. a low FB). In the case of a low fraction of birds, we expect a higher fraction of biotype *molestus*. Hence, we think that scenarios that involve a combination of a high FB and a low fraction of biotype *pipiens* or a low FB and high fraction of *pipiens* are not representative of a real situation. Therefore, we decided to present the 54 scenarios in six plots (with low and high FB and with the three mosquito population compositions). For each of these groups of scenarios, the estimated value for *R*_0_ is given for each possible combination of temperature and mosquito-to-host ratio *C*.

In addition to *R*_0_ calculations, elasticity analysis can be done on the NGM. By applying this analysis, which is a form of perturbation analysis, to the elements of the NGM, the contribution of each of the biotypes to the total can be quantified. This method has been applied in population biology for a long time^33^, but more recently, it has been used in vector-borne disease eco-epidemiology^34, 35^. For this analysis one calculates the proportional response in a dependent variable resulting from a proportional perturbation in an independent variable^36^. The interesting aspect for our application is that the elasticities of the dominant eigenvalue of a matrix to the matrix elements always sum to one^37^. In population biology, this has led to their interpretation as the relative contribution to population growth from the respective transition in the life cycle^33^. Similarly, the element elasticities of an NGM can be interpreted as contributions to *R*_0_. The relative contributions are given exactly by the elasticities. Composite elasticities, sums of particular sets of element elasticities, follow from the interpretation of element elasticities as contributions to population growth rate or *R*_0_^34, 35^. In this case, the two elements that refer to a specific biotype will always be the same. We, therefore, chose to plot the proportion of the transmission associated with each of the biotypes. Calculations of *R*_0_ were done based on the point estimates for each parameter (Table 1).

## Results

**Figure 2:**
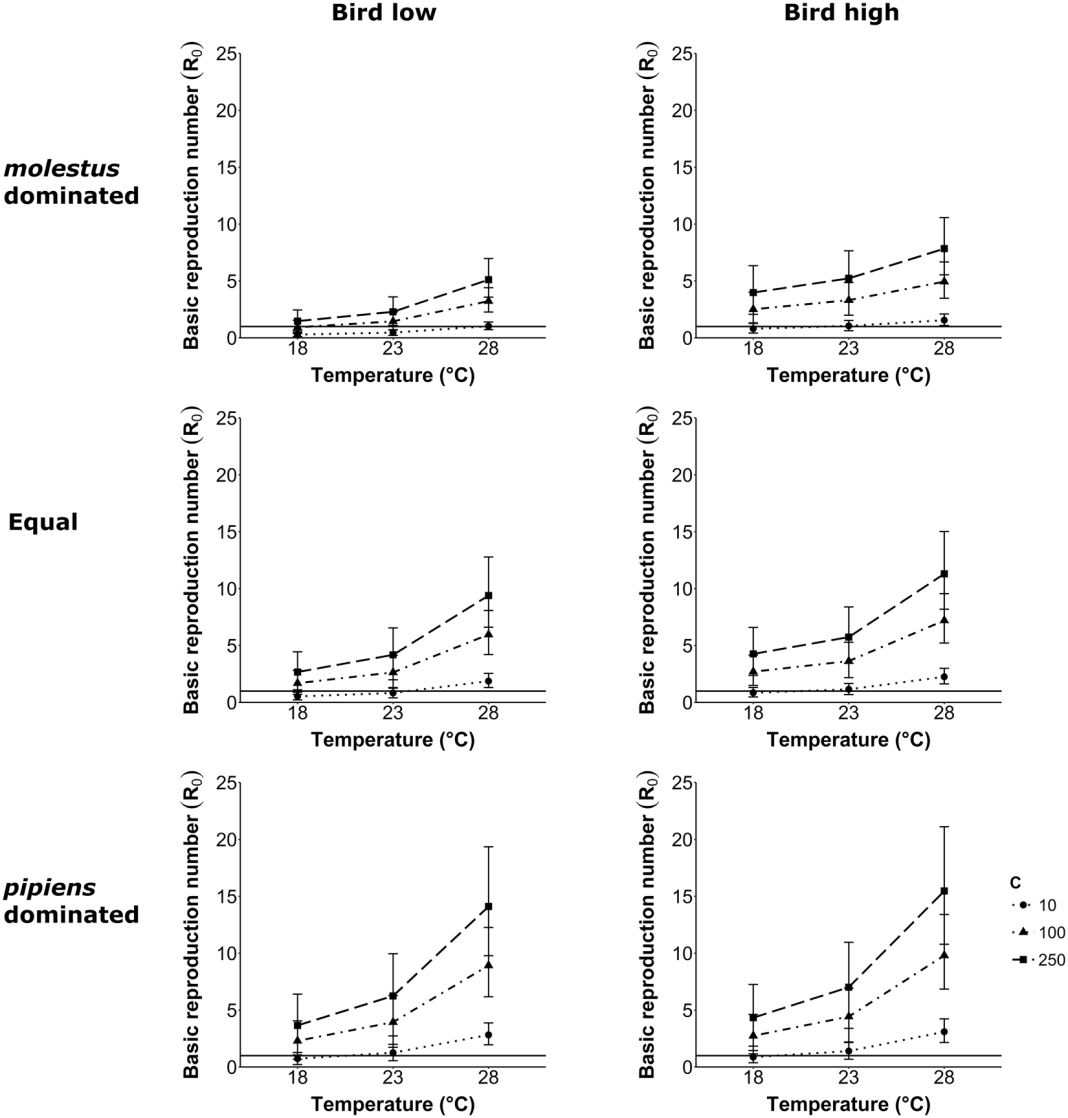
Scenarios for West Nile virus transmission risk modelled with parameters relevant for Europe. The six plots show the results for low or high fractions of birds in the host population (FB = 0.2 and FB = 0.8, respectively) and one of the three different mosquito population compositions (*molestus* dominated, equal fractions, or *pipiens* dominated). Each plot shows the mean *R*_0_ out of 100,000 model calculations, with error bars showing the 95% range of calculated *R*_0_ values for three temperature scenarios (T = 18 °C, 23 °C, and 28 °C) and three different mosquito-to-host ratios (*C* = 10, 100, or 250). The horizontal line indicates an *R*_0_ value of one. Values of *R*_0_ above one indicate that there is a chance of West Nile virus establishment.

In total, 54 scenarios were modelled in order to investigate the effects of temperature, bird fraction, mosquito-to-host ratio, and *Cx. pipiens* biotype composition on *R*_0_ (Fig. 2). The first observation is that there are considerable differences among the 54 different mean values of *R*_0_, ranging from values well below one to values of *R*_0_ of approximately fifteen. Temperature, mosquito-to-host ratio (*C*), population composition of hosts (bird fraction FB), and biotype composition are all important in determining the value of *R*_0_. Here we will describe the effects for each of the variables.

### Temperature

Within each of the six plots, *R*_0_ values increased with higher temperatures (Fig. 2). Our results suggest that under favourable climatic conditions with relatively high average temperatures of at least 28 °C, there is a high chance that WNV transmission cycles become established. Such temperatures are typical for southern European countries. The risk of WNV establishment seems, therefore, highly dependent on temperature and to a much lesser extent dependent on the exact mosquito population composition, bird fraction, and mosquito-to-host ratio. In contrast, *R*_0_ values were much lower at 18 °C, especially for scenarios with the lowest mosquito-to-host ratio (*C* = 10). This suggests that WNV establishment in regions with low average summer temperatures of 18 °C, such as northern Europe, seems only possible when a high number of mosquitoes is present per host. This confirms that temperature is one of the most important drivers for WNV transmission^38^, which can explain the differences in WNV transmission between northern and southern Europe.

### Mosquito-to-host ratio

In all six plots the *R*_0_ values increased when more mosquitoes are available per host (Fig. 2). Regions with on average at least 100 mosquitoes available per host, have an increased risk of WNV establishment because of relatively high *R*_0_ values at all temperatures. Thus, the mosquito-to-host ratio is a second important determinant for the risk of WNV establishment in a certain area.

### Fraction of birds

The difference in *R*_0_ values between the main scenarios with low and high bird fraction is relatively small (Fig. 2). This is linked to the fact that fraction of birds in the host population determines both the host preference of the biotypes as well as the distribution of the bites over the birds. High fractions of birds mean that all biotype *pipiens* and also a substantial part of the *molestus* and hybrid mosquitoes will feed on birds, but also that the bites will be distributed over more birds. Thus, the actual number of bites per bird decreases with higher fraction of birds, which results in fewer mosquitoes infected by one bird. However, the number of birds infected by one mosquito increases, as the preference shifts to birds with increasing FB. These two effects counteract each other, but overall, the *R*_0_ was slightly higher for the scenarios with high FB.

### Biotype composition

Biotype *pipiens* plays an important role in WNV transmission due to its preference for birds and relatively high vector competence, especially at 28 °C. With increasing fractions of biotype *pipiens* in the population, there is an increase in *R*_0_ values for all scenarios (Fig. 2). In the main scenarios with equal fractions of biotypes and their hybrids, there is also a relatively high risk of WNV establishment, especially when mosquito-to-host ratios are at a medium or high level. In such scenarios, there is a higher chance of spill-over to humans if WNV transmission can get established.

### Biotype contribution

**Figure 3:**
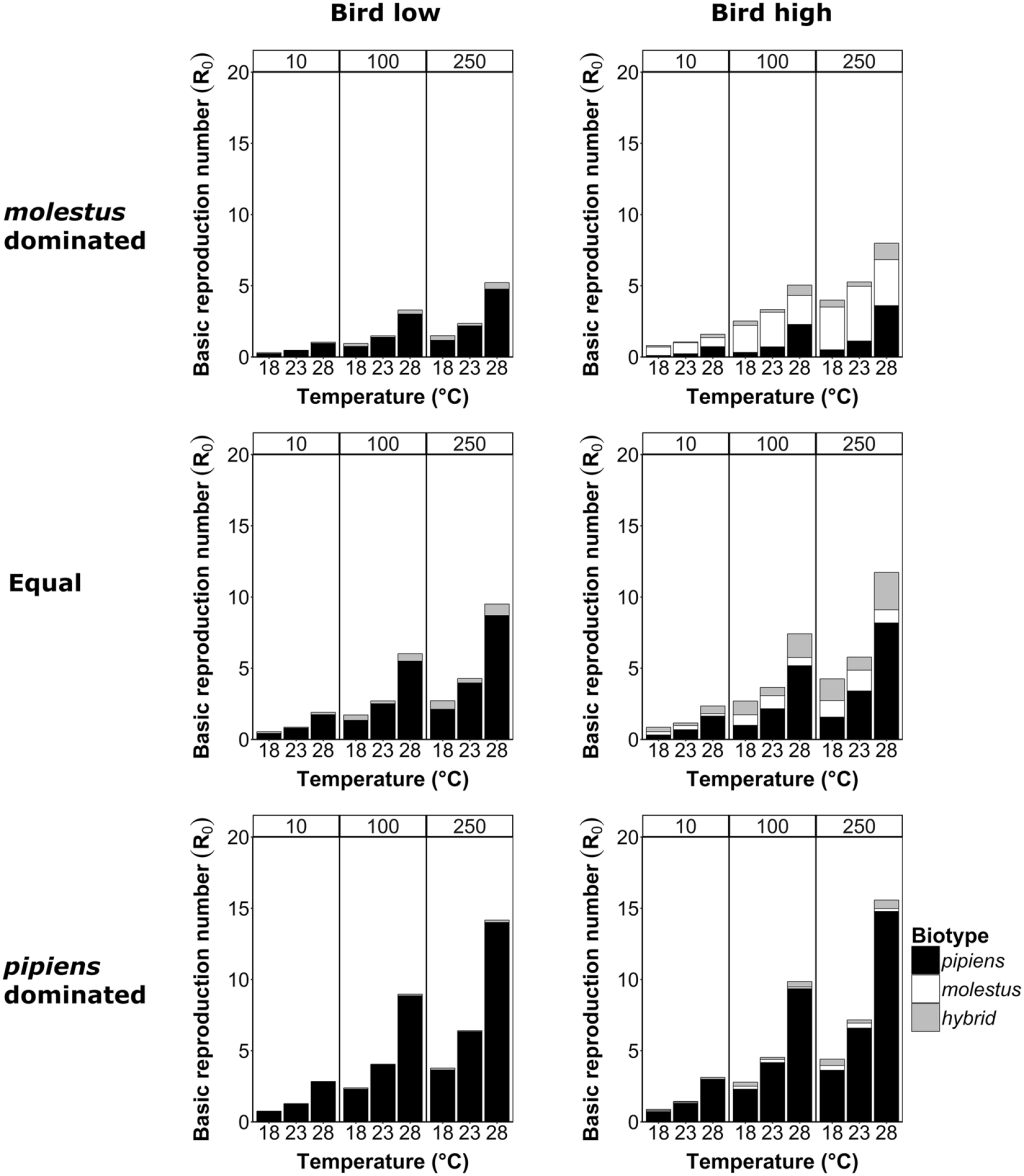
Contribution of each *Culex pipiens* biotype to scenarios for West Nile virus transmission in Europe. The six plots show the results for low or high fractions of birds in the host population (FB = 0.2 and FB = 0.8, respectively) and one of the three different mosquito population compositions (*molestus* dominated, equal fractions, or *pipiens* dominated). Each plot shows the contribution of biotype *pipiens*, biotype *molestus* or hybrids to the *R*_0_ values for three temperature scenarios (T = 18 °C, 23 °C, and 28 °C) and three different mosquito-to-host ratios (*C* = 10, 100, or 250). Values of *R*_0_ above one indicate that there is a chance of West Nile virus establishment.

In order to investigate the role of each *Cx. pipiens* biotype in WNV transmission, we performed elasiticity analyses to calculate the contribution of each biotype to the mean *R*_0_ (Fig. 3). At low bird fractions, biotype *molestus* and hybrids will mainly bite hosts other than birds, whereas biotype *pipiens* will still have a preference for birds. Therefore, in all three main scenarios with a low bird fraction, biotype *pipiens* is mainly responsible for WNV transmission. In contrast, when the fraction of birds increases the contributions of biotype *molestus* and hybrids also increase due to a shift in host acceptance towards birds (Fig. 1). In mosquito populations with high fractions of biotype *molestus*, they are the main contributor to *R*_0_ (Fig. 3). However, scenarios with both a high fraction of birds and a high fraction of biotype *molestus* are unlikely to occur in natural environments. Overall, hybrids may play a minor role in the initial phase of WNV transmission, but may become more important once WNV transmission cycles have established in a certain area. Presence of hybrids increases the risk of infection of humans with WNV.

## Discussion

The aim of this study was to explore the role of temperature-dependent and mosquito biotype-specific effects on the risk of WNV establishment, using laboratory and field-derived data. We show that various sets of parameters, comprising 54 scenarios, result in distinct *R*_0_ outcomes, which clearly demonstrates the complexity of the different parameters modelled in this study. Especially the interaction between temperature and biotype composition, and the mosquito-to-host ratio are main factors that influence *R*_0_, and, thus, the chance that WNV can get established in a certain area. In addition, *Cx. pipiens* biotypes contributed differentially to the *R*_0_ which underscores the necessity to include biotype-specific parameters in models for reliable WNV risk assessments.

Temperature does not only have a direct effect on viruses (e.g. increased viral replication at higher temperatures^39, 40^), but it also affects mosquitoes in several ways, such as decreased duration of the gonotrophic cycle, and increased biting rate at higher temperatures^41, 42^. These effects together result in higher vector competence for WNV at higher temperatures^18, 20^, which, in its turn, contributes to a higher risk of WNV establishment^43^. In this study, we chose representative average summer temperatures for northern (18 °C) and southern Europe (28 °C), as well as an intermediate temperature (23 °C)^44^. In all scenarios, temperatures of 28 °C allowed for establishment of WNV, which is in line with ongoing WNV circulation and outbreaks in European countries such as Italy, Hungary, and Greece^18, 45^. In contrast, WNV establishment is only likely at temperatures of 18 °C, if there is a high mosquito-to-host ratio. This shows that temperature is an important limiting factor for WNV transmission in northern Europe. More frequent and longer periods of intense heat in northern Europe due to global warming may, thus, coincide with establishment of WNV transmission cycles in northern Europe^46–49^.

WNV transmission risk is not only influenced by temperature, but also by several other environmental factors, such as rainfall and humidity^49–51^. Depending on local circumstances, rainfall and humidity can have positive as well as negative effects on WNV transmission risk^38, 52, 53^. Therefore, these effects are difficult to incorporate in the model. However, since most of the impact of rainfall and humidity will be via their effects on mosquito population density, we can consider these effect indirectly by looking at the mosquito-to-host ratio *C*. By considering different values for this parameter, we have indirectly taken different scenarios for favourable (high *C*) and less favourable circumstances (low *C*) for mosquitoes in terms of rainfall and humidity into account.

The rationale and assumptions underlying our model are similar to the ones of the traditional Ross-Macdonald model equation^54–56^. The main difference is that the next-generation approach allows us to include multiple types of infectious individuals, in this case different biotypes. The EIP is assumed to take a fixed number of days and the biting and mortality rates are constant (that is, the times to the next bite and to death are exponentially distributed). This set of assumptions, with a fixed duration for the EIP, might give a lower survival of the EIP than other assumptions^26^, which means that our estimates for *R*_0_ are likely to be conservative. However, as we selected a broad range of parameter in diverse scenarios, the overall range in *R*_0_ values is still relatively large.

In our model, we did not include vertical transmission from mosquito to mosquito, or bird to bird. However, vertical transmission may contribute to WNV transmission in the field, and can especially be key for overwintering of WNV in vertically infected mosquitoes^57, 58^. Consequently, outcomes of the *R*_0_ may slightly increase if vertical transmission would be included.

The mosquito-to-host ratio is an important determinant of the value of *R*_0_^59, 60^. The underlying assumption for including the mosquito-to-host ratio in the *R*_0_ formula is that the mosquitoes in a certain area will distribute their bites over the hosts available in that area and that the mosquito-to-host ratio thus determines the number of bites that a host will receive. The number of bites received by a host is assumed to be the number of mosquitoes searching for a blood meal (number of mosquitoes multiplied by the probability they will feed on a certain day) divided by the number of hosts. This assumption is perfectly plausible in a small confined space: the mosquito bites are likely to be distributed over the available hosts. However, caution should be taken when modelling host and vector abundance separately, or when varying the mosquito-to-host ratio, as we did in this study by varying the value of *C*. The consequence of this assumption is that the *R*_0_ estimate decreases with higher host densities (more hosts, so fewer bites per host, resulting in lower *R*_0_ values). This is counterintuitive and in most cases probably incorrect, since densities of vectors and hosts are not independent. Since vectors depend on hosts for blood meals it is unlikely that high abundances of vectors can persist in an area with few hosts, or vice versa that areas with many hosts would harbour only few vectors. Also, as distance between hosts is not taken into account, the fact that lower host densities actually may hamper the spread or reduce the chance to find another host, is not taken into account. This issue explains why the fraction of birds did not seem to affect the risk of WNV transmission in our models: at low values of FB, many *pipiens* would already bite on birds and at higher values of FB, the increase in number of bites would not be very substantial. But at a high FB, the number of birds would be higher, and hence the number of bites would be ‘spread out’ over more birds, which would lead to fewer bites per bird and hence fewer opportunities for a bird to pass on the infection. As stated above, this assumption may not always be realistic, as vector and host densities are not independent. We tried to at least partly overcome this issue by focusing on the more plausible scenarios in terms of the fractions of birds and the mosquito population. We also refrain from drawing strong conclusions on the effect of varying the fraction of birds or the mosquito-host-ratio itself, as these two variables, together with the mosquito population composition and the host preference, are strongly interacting in the way they determine the number of bites per bird. We, therefore, argue that field studies focussing on the relationship between these variables and the number of bites received by individual hosts would be very important in improving the assessment of risk of mosquito-borne diseases.

The elasticity analysis showed that biotypes contributed very differently to *R*_0_. These differences are due to the relative abundance of biotypes, biotype-specific behaviour (especially host preference^14^), and vector competence^20^. Biotype *pipiens* plays an important role in establishment of WNV transmission, due to its preference for birds and relatively high vector competence at 28 °C. Biotype *molestus* and hybrids contribute remarkably less to WNV establishment, especially in areas with low bird occurrence, because they will readily feed on the more abundant mammalian hosts. However, a shift in feeding behaviour has been observed for biotype *molestus* in areas with high bird abundance^61^. Such shifting feeding behaviour would increase the contribution of both biotype *molestus* and hybrids to WNV transmission. More importantly, feeding on both birds and mammals increases the risk that WNV will be bridged from birds to humans and horses. Thus, differentiating between biotypes and including relevant parameters in *R*_0_ models is essential for accurate WNV risks assessments.

*R*_0_ models are a powerful tool to assess risks of disease establishment in certain areas. The next-generation matrix approach allows for inclusion of different type-at-infections and here, we used this property to develop a novel *R*_0_ model that incorporates the different *Cx. pipiens* biotypes, their feeding preferences and different response of their vector competence to temperature. We have shown the importance of including these temperature-dependent and biotype-specific effects as the *R*_0_ values varied substantially between the different scenarios. This stresses the importance of biotype differentiation in entomological surveillance programmes, and of including temperature and biotype-specific parameters in risk assessments.

## Conclusions

The interaction between temperature and the *Cx. pipiens* biotypes, and the mosquito-to-host ratio are important factors that determine the chances of WNV establishment in a certain region. Currently, temperature is an important limiting factor for WNV circulation in northern Europe, but the modelled scenarios show that transmission cannot be ruled out. Accurate data are needed on how the number of bites received by birds varies with mosquito and host abundances, and on how these abundances may vary across habitats. Different contributions of the *Cx. pipiens* biotypes to the *R*_0_ highlight the importance of determining biotype-specific parameters in models for reliable WNV risk assessments.
